# Aquaphotomics—Exploring Water Molecular Systems in Nature

**DOI:** 10.3390/molecules28062630

**Published:** 2023-03-14

**Authors:** Jelena Muncan, Roumiana Tsenkova

**Affiliations:** Aquaphotomics Research Department, Graduate School of Agricultural Science, Kobe University, Kobe 657-8501, Japan

Since its birth in 2005, when introduced by Prof. Roumiana Tsenkova, and especially in the last five years, the development of aquaphotomics has shown a rising trend. The increasing interest in using aquaphotomics in scientific research can be observed by a rough analysis of the data provided by the Google Scholar search engine (Figure 1). This simple query for the published papers and patents (without citations of those works) on a year-by-year level since the establishment of aquaphotomics in 2005 shows that the slow, steady growth of the first decade is now being replaced with a more rapid trend, with publications doubling in numbers every two years.

A similar query performed using Web of Science provided more information about the categories of published scientific articles which featuring the word “aquaphotomics” [1]; there were 25 (Figure 2). The two categories to which the majority of the published works belong to are multidisciplinary chemistry (23.81%) and analytical chemistry (20.00%), which together comprise almost half of all the results, followed by biochemistry/molecular biology (19.05%) and spectroscopy (18.10%). Out of the 25 found categories, 4 contain the words “multidisciplinary”, 2 “inter-disciplinary”, and 3 “applied” or “applications”.

This “character” of aquaphotomics as an inter-disciplinary field with lots of applications across many disciplines is well-reflected in this book, which in one place collected high-quality, original research articles and reviews that were originally published as open access articles in a Special Issue of Molecules titled “Aquaphotomics—Exploring Water Molecular Systems in Nature”.

Out of the 21 published articles, 3 were reviews [2,3,4] and 18 were original research papers [5,6,7,8,9,10,11,12,13,14,15,16,17,18,19,20,21,22], that can be roughly separated into 6 categories (Figure 3).

The majority of the articles, approximately one third, belongs to the field of applications in agriculture and food science [8,9,12,13,14,21,22], a field from which aquaphotomics originated, and probably the reason why this category is still at the forefront of aquaphotomics development. Following this category are the preprocessing and chemometrics [2,4,15,18] and fundamental studies [5,6,10,17]. The former category reflects an important issue that aquaphotomics is trying to resolve, namely, the necessity of developing appropriate tools for dealing with the spectra of highly aqueous samples, and that significant efforts are being invested in resolving the issues of extracting the information from very subtle spectral signals. The papers that can be classified as belonging to the Fundamental studies show a recognition of aquaphotomics as a novel tool that can provide new insights into many puzzling phenomena in nature where water plays the role. They also show the possibility of opening new research directions and novel fields of applications.

This is exactly something that can be said about the category of material science [7,16], a relatively new area where aquaphotomics is slowly starting to make progress. The last two categories, cell biology [3,20] and diagnostics and therapy [11,19], do not feature many research papers because of the difficulty of conducting research with living systems. However, these two categories, and perhaps life science in general, still represent an important direction of growth that holds the strongest potential for making an impact and lasting change in our world, by transforming the current practices and offering non-destructive, real-time monitoring and early diagnostics that hopefully one day will be replaced with prevention.

Another interesting aspect of the original research articles is the measurement methods the researchers employed (Figure 4). Contrary to expectations that this would be the major portion, only four of the original research papers reported results obtained by using near-infrared (NIR) spectroscopy in the area of the first overtone of water (1300–1600 nm) [5,6,19,22].

The use of only the first overtone of water in analysis has been the dominant choice in aquaphotomics publications for a long time. This is because aquaphotomics relies on specific water bands as water matrix coordinates (WAMACs), which have been well-defined through studies in this region. As a result, there is now a clear understanding of the assignments of these water absorbance bands and their relation to water functionality. Nevertheless, it is very encouraging to notice that the use of short wavelength NIR spectroscopy is gaining popularity. In this Issue, three papers reported the use of short wavelength NIR spectroscopy, in the approximate range of 750–1000 nm, where the 2nd and 3rd overtone of water are located [9,13,21]. Furthermore, two research groups presented the results, in parallel, for both the first overtone of water region and higher overtones, demonstrating increased efforts in gaining more clarity in the nature of information that can be gained from each of the regions, and attempts at defining WAMACs similar to the existing ones in the first overtone [23]. Additionally, three studies were performed over the entire (~750–2500 nm) [16,18], or nearly the entire range, of the NIR spectrum (1100–2500 nm) [15]. This testifies to increased interest in the water spectral features over the entire range, which may result in many benefits for both scientific and practical aspects.

It is especially encouraging to witness that the light–water interaction over the whole electromagnetic spectrum is a source of valuable information, as stated in aquaphotomics. One paper reported the results in the mid-infrared region [7], and two used Raman spectroscopy [10,17]. There were also two papers reporting on the exploration of a newly developed measurement technique that employs GHz sensors [20], and also perturbation by polarization filters [13], for the first time in aquaphotomics. Studies such as those challenge the current understanding of what exactly is the nature of light–water interaction and bring attention to the importance of the design of instruments and the choice of light, which is a measurement and a perturbation tool in aquaphotomics.

In this book, all the papers from the Special Issue are organized to follow the trend from fundamentally important topics from which all the readers can benefit, through the rising complexity of the aqueous and biological systems that were investigated. That is why the beginning of the book presents papers dealing with the topics of preprocessing, chemometrics, and the related tools that should lead to improved ways of extracting the relevant information from the spectra. In the first review of this Issue, Roger, Mallet, and Marini [2] provided a comprehensive overview of the most useful preprocessing and chemometrics techniques that are particularly relevant for the nature of signals aquaphotomics is dealing with. If one should choose just one, the most important message of this review should be as follows: “It is important to look at the “raw” spectra before deciding which preprocessing to use!”. This is something that cannot be emphasized enough. It is a common mistake in novices to rush and apply chemometrics or create aquagrams according to the explanations provided in the literature [24,25] and jump to interpretations based on the aquagram only. However, this review, through three examples of data, show how inadequate preprocessing, and overlooking the critical step in seeing the spectra before creating aquagrams, can result in a distorted view and misinterpretations. It is our expectation that this review and the techniques proposed are going to be of major significance for future aquaphotomics studies, and readers and researchers are strongly encouraged to start exploring and using the proposed preprocessing techniques. The review paper by Sun, Cai, and Shao [4] summarized novel findings regarding the extraction of structural and quantitative information from the temperature-dependent NIR spectra using chemometrics methods. Shao and his team are leaders in the development of temperature-dependent spectroscopy [26,27,28,29,30,31,32], where they utilize the phenomena of using the water spectral changes with temperature when the system undergoes changes in composition. In these applications, water plays the role of a “scanner”, acquiring information through the changes in its structure caused by the changes in concentration. The current paper, in addition to providing an overview of chemometrics techniques that can be used in temperature-dependent NIR spectroscopy, highlighted also some of the associated challenges, such as the need for a large number of reference samples and difficulties in finding the most appropriate chemometrics method.

In her work, Cui tackled one of the major issues that can happen in both research and industrial settings: the need to correct the spectral variations caused by the different instruments, measurements, and sample conditions [15]. This problem is of particular importance for all aquaphotomics studies because it uses the high variability of aqueous samples due the very nature of hydrogen bonding in the water molecular network and the water response to even subtle changes either in its own composition or in the environment, and the accuracy and uniformity of the instruments are very important. Cui proposed a newly developed algorithm, called mutual-individual factor analysis (MIFA), which takes advantage of the sensitivity of water to perturbations, to divide the water spectral region into mutual and individual parts, which derive from the spectral features among instruments (mutual) and differences in the sample and measurement conditions (individual). The effectiveness of the newly developed MIFA algorithm for the calibration transfer was successfully demonstrated on two datasets of corn and wheat, showing a novel solution for the standardization of spectra derived from different instruments when measurements are affected by multiple factors.

In one of the very innovative and creative works, Ye et al. developed a new discriminating analysis for the purpose of application in problems of the classification and identification of water samples with small differences in the component content; this is a very hot topic, especially in the quality control of drinking water [18]. The work is rich in novelty in many aspects: they utilized the optical path length of cuvette as a perturbation factor, performed fusion modeling on multi-optical path measurements, and developed a new pattern recognition method that effectively discriminated between three types of drinking water with a very similar content. The novel approach could widely be applied to other types of samples or in other fields.

When it comes to studies that reveal some fundamental new insights, the work by Stoilov et al. [6] stands out, reporting for the first time that the frequency of applied sound perturbation results in distinctive changes in water’s molecular structure, that was captured in a water absorbance spectral pattern. The team which investigated two types of water concluded that perturbation by sound produces a measurable impact on water’s molecular structure which depends on the nature of the water samples used, as well as the frequency at which at the sound is tuned to. Similarly, very novel results are reported by Muncan et al. [5], who investigated how the water used in mixing for cement production influences the mechanical properties of the final products. The study showed that differences in water defined the properties of cement mortar very early, within the first 24 h. All the waters used in the study satisfy all the conditions as defined by current standards, but contrary to the expectations, they result in very different properties of the final products, showing the need for the recognition of water’s molecular structure as one of the aspects that needs to be taken into consideration and incorporated into future practices. Another study showing the importance of the knowledge of water’s molecular structure in order to predict the material properties is a study conducted by Maggiore, Tommasini, and Ossi [10]. The study was focused on using Raman spectroscopy to measure the amount of liquid water in snow. In snow, the water can exist in three phases simultaneously, solid, liquid, and vapor, but the content of liquid water is important to estimate snowmelt runoff and wet avalanche risk. This paper is another fascinating example of how the structure of water defines the mechanical properties of the material which, in this case, is snow. By reproducing the water percolation in snow in laboratory settings and monitoring the presence of liquid water using the Raman spectral features of ice and liquid water, the researchers were able to detect the presence of liquid water in a snow volume and to follow the evolution of the progressive filling of snow with the liquid phase in a non-destructive and minimally invasive optical approach. They also proposed the measures needed to upgrade the measurements from laboratory to on-field applications.

The study by Miwa et al. [17] used a similar technique: low-frequency Raman spectroscopy (which is a type of Terahertz spectroscopy) to study the phenomena of semi-clathrate hydrates, which are very popular materials with a wide range of applications due to their ability to store heat. The temperature at which they can store heat can be changed depending on the selection of certain guest-ions, and it is influenced by their size, hydrophilicity, crystal structure, and other properties. Employing low-frequency Raman spectroscopy, the team were able to observe interactions between the ions in semi-clathrate hydrates and found that the temperature at which they stored heat was related to the interactions between the ions, as shown by the position of peaks in the low-frequency Raman spectra.

Interesting and very novel papers are emerging from the area of material science, where for a long time, mid-infrared spectroscopy and Raman spectroscopy were dominant. Moll et al. [16], in contrast, focused on the near-infrared spectroscopic exploration of water in polymers with varying hydrophobicity. One of the most interesting findings of this study is that the changes in the NIR spectrum of water can be observed even in the interaction with highly hydrophobic polymers, leading the authors to conclude that hydrophilicity is not an exhaustive enough parameter to fully account for the interaction of a polymer with water. This also confirms one of the aquaphotomics postulates regarding the sensitivity of water towards its chemical environment and that it is so clearly manifested in the NIR spectra.

Meada et al. [7] employed mid-infrared spectroscopy to study polymers, but their study was performed from an unusual angle: they investigated vicinal water in soft contact lenses. The water in the vicinity of material surfaces has a significant impact on a variety of phenomena stemming from the interfaces, such as chemical reactions, adsorption, friction, adhesion, and biocompatibility. In the paper, the authors devised and proposed a new method to study the behavior of water near polymer materials using conventional ATR infrared spectroscopy. The method involves the application of pressure to a hydrated contact lens, which results in a deformation and change in the ratio of bulk and vicinal water. The spectral signature of vicinal water was then extracted using multivariate curve resolution–alternating least squares (MCR-ALS) analysis. The results showed that the shape of the OH-stretching band in the infrared region, which reflects the hydrogen bonding state of vicinal water, is different depending on the chemical structure of the polymers constituting the contact lenses. This method is also proposed to probe the hydration states of other materials whose function requires water, such as porous materials, hollow fibers, and other soft materials.

In the agricultural sector, aquaphotomics development in the area of food adulteration seem to be increasingly popular. Three papers in the Special Issue tackled challenges in food adulteration, specifically honey [8,21] and coffee [14]. While honey adulteration was a subject of investigations in several previous publications [33,34,35], the current ones contain two novelties. First, a study by Bodor et al. [14] for the first time reports the aquaphotomics results in the detection of honey adulteration by heat processing. Their results prove that even at a low temperature treatment (40 °C), measurable changes occur in the spectra of the honey, as demonstrated in aquagrams of three types of honey (sunflower, bastard indigo, and acacia), and that these spectral changes are mainly related to the transformation of the highly bonded water to less-bonded water or free water. Raypah et al. [21] investigated the potential of aquaphotomics for honey adulteration, but for the first time in a stingless bee honey, which is made of the nectar of trees, not flowers, and typically has a higher moisture content. Another novelty is the employment, for the first time, of short NIR spectroscopy and the detection of three types of adulterants: water, apple cider, and fructose syrup. This team presented a simple aquagram-based method for the detection of adulteration in the region 800–1100 nm, a very convenient region, where the majority of the most common photodetectors available on the market offer the best sensitivity. Another important contribution of this paper is in the assignments and interpretation of the absorbance bands found to be important for honey adulteration, and their importance from the standpoint of honey functionality. The coffee adulteration work by Balkis et al. [14] is another achievement in food adulteration detection, a particular research topic for which the group of Prof. Kovacs from Hungary is becoming increasingly recognized for. The coffee adulteration work was able to accurately determine the ratio of coffees Robusta (less expensive and easier to cultivate; therefore, commonly used as adulterant in higher-price coffee blends [36,37]) to Arabica in both ground and liquid forms. A classification accuracy of 100% was achieved for pure Arabica and Robusta, and the prediction of the Robusta to Arabica ratio occurred with an error of 2.4%. The aquaphotomics approach provided typical spectral fingerprints for each coffee blend and was able to accurately discriminate and estimate the Robusta content of marketed blends.

Marinoni et al.’s study [22], which focused on utilizing the potential of aquaphotomics to assess the freshness of ready-to-eat rocket salad, may mark the initiation of a promising new realm of applications. The work employed several techniques, including a portable E-nose, the electrolyte leakage test, NIR spectroscopy, and aquaphotomics to evaluate and monitor changes during storage of salad in three types of modified atmosphere at 4 °C. Although preliminary, the results suggest the potential of NIR spectroscopy to provide the modeling of the shelf-life of stored products, the identification of the most suitable atmosphere for maintaining freshness, and increasing insights into the role of water in the preservation/deterioration of fresh vegetables. In this study, in particular, the author found importance of water solvation shells for the description of time-related changes during storage.

Kaur et al. [12] performed an aquaphotomics study aimed at understanding the changes in the water structure of kiwifruit juice with changes in temperature. This study was performed in a style typical for Kaur, where she was focused on one very practical application point of aquaphotomics, the identification of problems and overcoming them by developing new tools and solutions, and, additionally, the study provided new information regarding the water spectral features and assignments, adding to the increasing aquaphotome database. This particular work identified that the influence of an increasing temperature on the peak absorbance of kiwifruit juice spectra manifests as a lateral (wavelength) shift in the first overtone and a vertical shift in the second overtone region of water. Further, experimenting with different preprocessing techniques (orthogonalization and extended multiplicative scatter correction), a temperature-independent partial least square regression model for predicting the soluble solids concentration (SSC) of kiwifruit juice were built for both overtones of water, significantly reducing the prediction bias. Additionally, most importantly, the approach employed in this work, may be applied to other problems such as the prediction of various properties of other fruit juices, intact fruit, or other types of samples, whenever robustness against temperature changes is desirable.

One highly novel work came from Rajkumar et al. [13], who used NIR spectroscopy and aquaphotomics to assess the spectral changes between linearly polarized and unpolarized light in commercially grown yellow-fleshed kiwifruit. Measurements were taken on both unpeeled and peeled kiwifruit using a handheld NIR instrument. The results showed that linearly polarized light activated more free water states, while unpolarized light activated more bound water states. These differences were attributed to the surface layers of the fruit. The soluble solid content (SSC) in the fruit was not a factor in these results as the aquagrams generated for SSC were similar for all configurations. However, within the aquaphotomics framework, it is a significant finding that bound water absorbed more unpolarized light than polarized light, which calls into question the nature of the light–water interaction and how the light properties or the way light is administrated to the sample actually influences the results of the measurements and modeling of the properties of interest. The authors provided a plausible possible explanation that differences are due to polarization sensitive structures, particularly in the near surface layers, but further work is needed and encouraged in this direction.

Only one of the papers in this Special Issue was devoted to plant physiology, using aquaphotomics to explore the cold stress response in five different cultivars of soybean that were engineered to encompass varying levels of tolerance to cold stress [9]. The study showed that all soybean cultivars have a different water molecular structure in the leaves when the plants are exposed to even mild stress conditions, which is very easy to detect very early. Specific water molecular structures in the leaves of soybean cultivars were found to be highly sensitive to the temperature, showing their crucial role in the cold stress response. Further, the study revealed differences among genetically modified cultivars, suggesting that the genetic modification is actually aimed at the end at achieving a specific water molecular structure in the leaves which is more stable in the conditions of temperature change.

Two more papers of this Special Issue report interesting new theories and findings in cell biology. First, the review paper by Disalvo et al. [3] focused on the role of water in biomembranes, the structures commonly thought as surrounding and protecting the cells. In this review, the authors strongly and persuasively present evidence that water should be considered a structural and thermodynamic component of membranes and suggests incorporating this into current theories about the role of membranes in cells. The idea is that membranes should be seen as open and responsive systems that are affected by metabolic events and changes in water. The authors also suggest that the relationship between water and the membranes should be considered in terms of free energy and other thermodynamic properties, which play a role in the behavior of these crowded systems. Shiraga et al. [20], in their original research paper, further experimentally confirmed some of these aspects. They employed a newly developed measurement system based on a near-field CMOS dielectric sensor operating at 65 GHz, that enabled measurements of the bulk water content in cells with a high precision and single cell resolution. The system was used to evaluate the changes in the bulk water content during the process of cell death in keratinocytes. The results showed that there was a significant increase in the bulk water content approximately 1 h before the membrane disruption, suggesting that the calcium flux may play a role in triggering the increase in water content.

Santos-Rivera et al. [11] conducted a pioneering work, using aquaphotomics for the rapid detection of bovine respiratory syncytial virus (BRSV) infection in cattle, based on the spectra of exhaled breath condensate. This is the first report on utilizing this type of sample to perform diagnosis that holds immense promise, especially for screening purposes in human population. It is worth noting that BRSV is a very contagious viral disease spread by aerosols and via contact between animals. The results showed that changes in the composition of the exhaled breath condensate during infection could be accurately differentiated from the pre-infection stage with an accuracy of over 93%. These findings suggest that NIR aquaphotomics could be used to develop a non-invasive, in-field diagnostic tool for detecting not only BRSV infection in cattle, but similar infections in the human population.

In a very interesting case study, Scholkmann and Tsenkova [19] used aquaphotomics NIR spectroscopy as a tool to monitor the effects of a blood cleaning treatment called double-filtration plasmapheresis (DFPP). The analysis of the spectra acquired from a hand of one subject subjected to DFFP treatment showed that the water properties in the tissue changed after the treatment, characterized by an increase in small water clusters, free water molecules, and a decrease in hydroxylated water and superoxides. The changes in tissue water suggest that the positive effects of DFPP may be linked to improvements in the water quality in blood and tissues, related to the respective water molecular structures. This study is the first to document these changes after DFPP treatment in human tissue, and it is worth noting the existence of similarities between this study and some preliminary findings reported about the hemodialysis and the blood filtration treatment, namely, a marked increase in free water molecules after the filtration [38].

The works performed by the researchers who authored the articles in the Special Issue are all at the cutting-edge of aquaphotomics. Many of the works are the first of their kind, pioneering in nature and showcasing the new phenomena explored through the prism of water–light interaction. However, when one finishes the reading, it becomes apparent that some questions are still left without certain answers, and, furthermore, that new questions have emerged. It is now down to us to encourage others to be stimulated by these new questions, take this challenge, and have courage to create new paths into as of yet unexplored lands.

## Figures and Tables

**Figure 1:**
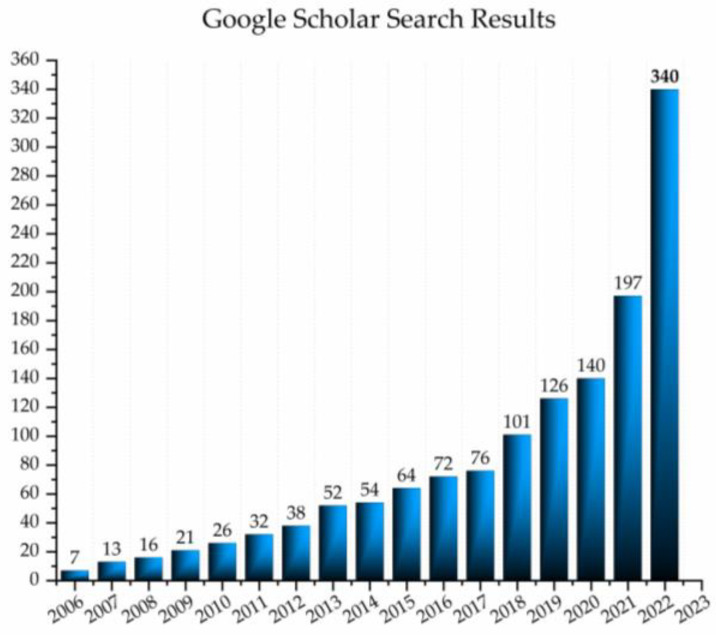
The results of analysis using Google Scholar search tool for articles and patents (excluding citations) mentioning word “aquaphotomics” since its establishment until 2022.

**Figure 2:**
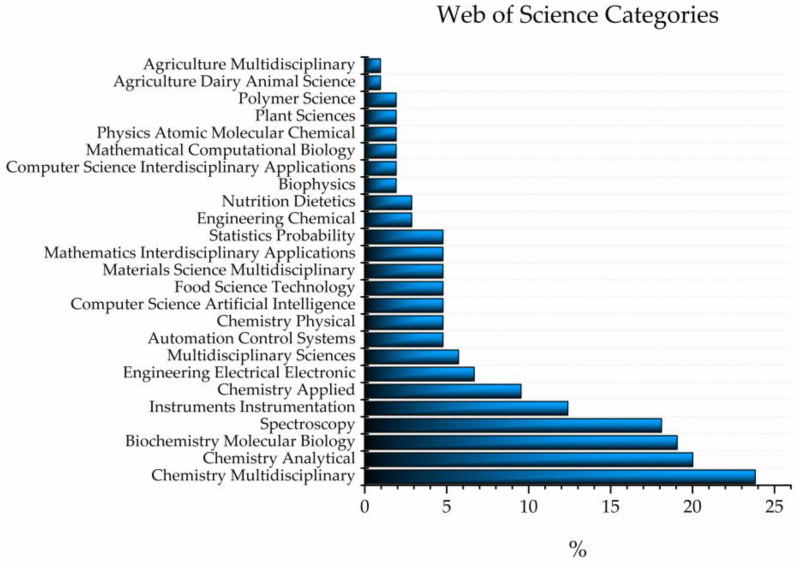
The results of Web of Science search for documents containing word “aquaphotomics” sorted according to categories defined by Web of Science. The majority of results (almost 50%) can belong to the categories of multidisciplinary chemistry and analytical chemistry.

**Figure 3:**
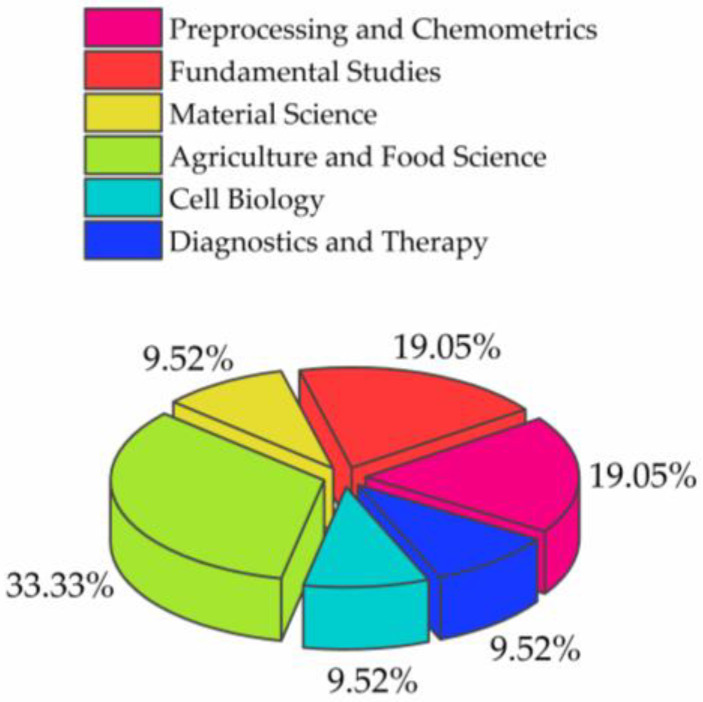
The main categories of scientific articles published in the Special Issue “Aquaphotomics—Exploring Water Molecular Systems in Nature”.

**Figure 4:**
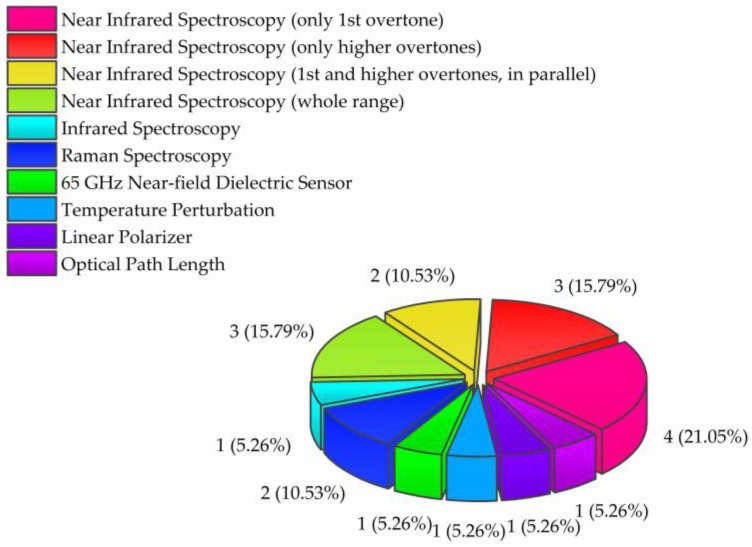
The types of measurement methods and perturbation techniques used by researchers in original research articles of the Special Issue “Aquaphotomics—Exploring Water Molecular Systems in Nature”. The numbers beside each category indicate the total number of papers that used a specific measurement method, while the numbers in parentheses represent the percentage of the total number of publications in the Special Issue.
